# Bayesian Sequential Pragmatic Cluster Randomized Clinical Trial Design for PrEventive Effect of MEditerranean Diet in Children: PEMED Trial Research Protocol

**DOI:** 10.3390/jcm14010240

**Published:** 2025-01-03

**Authors:** Danila Azzolina, Salvatore Auricchio, Luigi Greco, Renata Auricchio

**Affiliations:** 1 Department of Preventive and Environmental Science, University of Ferrara, 44121 Ferrara, Italy; 2 Clinical Trial and Biostatistics, Research and Development Unit, University Hospital of Ferrara, 44121 Ferrara, Italy; 3 European Laboratory for Food Induced Diseases, University of Naples Federico II, 80131 Naples, Italy; salauric@unina.it (S.A.); ydongre@unina.it (L.G.); r.auricchio@unina.it (R.A.)

**Keywords:** children, mediterranean diet, cluster-randomized, bayesian, sequential trial

## Abstract

**Background:** Childhood nutrition plays an important role in the promotion of long-term health. Introducing solid foods in alignment with the Mediterranean Diet during weaning fosters a preference for healthy foods early in life. However, access to nutritious diets remains a challenge in underserved communities. Scampia, a socioeconomically disadvantaged district in Naples, Italy, exemplifies a community where barriers to healthy eating persist. This research reports a trial protocol that plans for a study to evaluate the impact of the Mediterranean Diet on child health and to establish preventive strategies for chronic diseases. **Methods:** The PEMED (PrEventive effect of MEditerranean Diet in Children) trial is a Bayesian Sequential Pragmatic Cluster Randomized Clinical Trial. Family Pediatricians (FPs) are randomized to deliver either Mediterranean Diet-based dietary guidance starting at weaning or standard dietary practices using typical baby foods. Children will be followed up for six years, with regular assessments of growth, microbiome composition, and adherence to the Mediterranean Diet, using validated tools. Interim analyses will be conducted at three-year intervals to evaluate the efficacy and monitor adverse events. Saliva and stool samples will be collected for genetic and microbiome analyses, and adherence will be monitored through quarterly dietary recalls and biomarkers. **Results:** This trial will consider Italy’s established FP network for implementing innovative dietary intervention in a real-world setting. **Conclusions:** This study will address nutritional disparities in the underserved Scampia community and provide a scalable model for early dietary interventions. The results will shed light on the role of the Mediterranean Diet in improving childhood health and informing public health strategies globally.

## 1. Introduction

The widely accepted link between systemic chronic inflammation and chronic diseases, such as obesity, diabetes, cancer, and autoimmune diseases, necessitates preventive strategies, owing to the diseases economic impact on health systems [1,2]. Diet and intestinal dysbiosis are important factors in chronic inflammation, and dietary components play a direct role in chronic disease pathogenesis by influencing microbiota composition [3,4]. The Mediterranean Diet is recognized not only as an anti-inflammatory nutritional program but also for its positive effects on the gut microbiota; it offers a food-based approach to prevent and treat chronic inflammatory diseases [5]. Moreover, recent studies have provided evidence for the benefits of the Mediterranean Diet on cardiovascular health, which includes a reduction in the incidence of cardiovascular outcomes and risk factors such as obesity, hypertension, metabolic syndrome, and dyslipidemia [6]. There is also evidence that the Mediterranean Diet is associated with lower rates of incident diabetes and better glycemic control in patients with diabetes, compared to those on control diets [7]. In prospective studies, adherence to the Mediterranean Diet reduced mortality, especially cardiovascular mortality, and hence increased longevity [8]. In addition, it has been associated with less age-related cognitive dysfunction and a lower incidence of neurodegenerative disorders, particularly Alzheimer’s disease [9].

While direct evidence in infants is limited, research supports the benefits of introducing nutrient-rich, diverse foods early in life, which aligns with Mediterranean Diet principles to promote long-term health. Moreover, pre- and perinatal taste exposure affects later food preferences [10,11], and practices such as baby-led weaning, which promotes early satiety responsiveness, may protect against obesity [12,13].

The benefits of the Mediterranean Diet are well-documented in adult populations [14] for conditions such as obesity, cardiovascular disease, and diabetes, but there is little evidence regarding its long-term effects when introduced during infancy. By focusing on this developmental period, we define a clinical trial protocol for obtaining novel results on how early-life adherence to the Mediterranean Diet can influence health outcomes, including dietary habits, microbiome composition, and the prevention of chronic diseases later in life [10,15,16,17].

Moreover, despite the Mediterranean Diet’s documented positive outcomes, adherence among children and adolescents is low [18,19,20], and there is a gap in intervention studies for children aged 0–3 years, particularly regarding the effects of early nutrition on food habits, Body Mass Index (BMI), and microbiota composition.

The central hypothesis of this study is that early and consistent exposure to the Mediterranean Diet, introduced at the weaning stage (5–6 months of age), will foster healthy eating habits, support microbiome development, and reduce the risk of childhood obesity and chronic diseases later in life.

In response to these pressing concerns, this study protocol proposes a Bayesian cluster randomized clinical trial, the PEMED (PrEventive Effect of Mediterranean Diet in Children) trial, to promote the Mediterranean Diet style of nutrition among children residing in Scampia.

The proposed study setting is the population residing in the 8th Municipality, Piscinola-Scampia, where the University Health Center of Federico II University, Italy, is located. Scampia is one of the most socioeconomically disadvantaged areas in Naples, with significant challenges such as high rates of poverty and unemployment as well as limited access to resources that promote healthy lifestyles [21]. These factors contribute to nutritional inequalities and poor health outcomes among residents, particularly children, which make it a critical area for targeted public health intervention [22]. The usual diet in Scampia, as in many socioeconomically disadvantaged areas, often deviates from the Mediterranean Diet [23]. It is characterized by a high intake of processed and convenient foods, low consumption of fresh fruits and vegetables, and limited access to high-quality proteins, such as fresh fish. Processed foods, often high in added sugars, unhealthy fats, and sodium, are calorie-dense but nutrient-poor. These contribute to childhood obesity, type 2 diabetes, and other metabolic disorders by displacing healthier options, such as fruits, vegetables, and whole grains [24]. For instance, data from the Italian National Institute of Statistics (ISTAT) highlight higher-than-average rates of childhood obesity in the Naples suburb and Campania region compared to the national averages (www.istat.it, accessed on 7 May 2024). With a resident population exceeding 92,000, this municipality provides a robust platform for conducting population-based pragmatic clinical trials [25].

This trial design is structured to harness the existing Italian Family Pediatrician (FP) system, which has been a cornerstone of healthcare delivery in Italy since 1983 [26]. FPs under the Italian National Health System provide care to nearly all children in the area. This established network facilitates efficient recruitment, monitoring, and implementation of the intervention while also ensuring that the study aligns with existing healthcare practices.

This manuscript presents the study protocol for the PEMED trial and adheres to the Standard Protocol Items: Recommendations for Interventional Trials (SPIRIT) guidelines (Supplementary Material). The planned trial design is a Bayesian adaptive sequential study; unlike traditional trial designs that rely on fixed sample sizes and rigid endpoints, this approach allows for the continual accumulation of data as the study progresses [27] and for real-time adjustments in response to emerging patterns and potentially leading to more efficient and informative results [28].

The primary objective of the planned trial is to evaluate the impact of early Mediterranean Diet exposure on children’s health outcomes, hypothesizing that consistent MD adherence improves growth, reduces chronic disease incidence, and promotes a healthier gut microbiome. The trial compares the MD intervention to local dietary practices, which are predominantly reliant on industrial baby foods, to assess its effectiveness on growth and disease prevention.

## 2. Materials and Methods

### 2.1. Study Design

#### 2.1.1. Study Setting

This study population is the population residing in the 8th Municipality, Piscinola-Scampia.

Scampia municipality has a total population of 92,600, of which 28,915 fall within the age group–0–18 (www.istat.it, accessed on 7 May 2024). Based on historical data (www.istat.it, accessed on 7 May 2024), approximately 1179 births per year are expected within this population for six years. Table 1 provides an overview of the expected prevalence and incidence rates of specific pediatric diseases in Piscinola-Scampia. Data were retrieved from official Italian statistics (www.istat.it, accessed on 7 May 2024). The study population consists of 28,915 children aged 0–18 years. To assess the prevalence, the table lists the rate per 1000 children for each condition. This rate allows for the calculation of expected prevalent cases by adjusting for total population size. For instance, obesity has a prevalence rate of 75 per 1000 children, resulting in approximately 2169 expected cases (calculated by multiplying the prevalence rate by the population and dividing it by 1000). Each condition followed a similar calculation to provide an estimate of the total burden of that condition within this group of nearly 29,000 children.

In addition to prevalence, the table includes data on the incidence or rate of new cases, specifically focusing on newborns. With 1179 newborns added to the population each year, the table estimates the annual incidence of each condition among this cohort as well as the cumulative incidence over six years (totaling 7074 newborns across this period). For example, obesity has an annual incidence of 88 new cases per year among newborns, leading to an expectation of approximately 528 new cases over six years.

The expected prevalence is expressed as cases per 1000 subjects. The third column shows the anticipated number of prevalent cases in this population. The fourth column presents the number of incident cases, representing new cases per year based on the birth rate specific to the municipality, which was calculated as 12.42 per 1000 births. The corresponding incident cases are listed in the fourth column for the 1179 newborns expected during each year of the study. Throughout the six-year project period, the cumulative number of cases can be aggregated, as illustrated in the last column.

Enrollment and eligibility criteria

The following inclusion and exclusion criteria are applied in this trial:

Inclusion Criteria

- Healthy newborns with birth weight greater than 2000 g.
- Residence in Municipality 8, Piscinola, Scampia.

Exclusion Criteria

- Newborns suffering from severe premature birth.
- Newborns with congenital malformations.
- Newborns affected by severe diseases will be excluded from the study. Examples of such conditions include congenital malformations (e.g., congenital heart defects and neural tube defects), severe prematurity with a birth weight of less than 2000 g, genetic syndromes such as Down syndrome, metabolic disorders like phenylketonuria, and other serious medical conditions requiring prolonged neonatal intensive care or specialized treatment.

In cases where infants exhibit failure to thrive, Family Pediatricians (FPs) will closely monitor growth and health parameters. Adjustments to the dietary plan will be made as needed, incorporating additional nutrient-dense and calorie-rich foods to address growth concerns while maintaining the core principles of the Mediterranean Diet. The health and well-being of the child will remain a top priority, with individualized dietary modifications implemented where necessary.

If dietary crossover occurs and infants are introduced to processed foods along with Mediterranean Diet-based options, this will be recorded during regular dietary assessments. Such deviations will be addressed through targeted parental counseling, emphasizing the importance of adherence to the Mediterranean Diet and offering practical strategies to minimize reliance on processed foods. The study design includes regular follow-ups with parents to ensure that they remain engaged and supported throughout the intervention.

Changes in residency could impact the follow-up and data collection. To address this, participants will be required to provide proof of residence in Municipality 8 (Piscinola-Scampia) at the time of enrollment, ensuring that they were initially part of the study area.

We will implement strategies to minimize the impact of this movement. Families that relocate within a manageable distance of 15 KM will still be followed by their assigned Family Pediatricians (FPs), who serve as consistent points of contact. If a family permanently relocates beyond the feasible follow-up range, endpoint data will be collected at the time of departure to ensure that their partial contribution is included in the final analysis.

#### 2.1.2. Baseline Data and Child and Maternal Factors

Baseline data on maternal and family characteristics, including maternal age, education, smoking habits, medication use during pregnancy, and family socioeconomic status, will be collected. This includes maternal health conditions (e.g., pre-pregnancy obesity, gestational diabetes, and history of intrauterine growth restriction [IUGR]), infant birth weight, and family dietary habits. Maternal and family dietary practices will be assessed using structured questionnaires, while maternal health data will be obtained through medical records and self-reports.

The child and maternal factors considered will be as follows:

- Maternal and Family Variables: These will be collected at baseline to account for potential confounders in statistical analysis.
- Physical Activity: these factors will be measured during follow-up visits to evaluate their role in influencing child health outcomes.
- Breastfeeding Duration: This will be assessed both at baseline and during follow-ups to capture its influence over time.

The details of these variables are reported in the Table 2.

#### 2.1.3. Consent and Randomization

In this study, Family Pediatricians (FP) will be randomized to either the treatment arm (Mediterranean Diet) or the control arm (conventional care delivered to pediatric patients). The FP will deliver interventions to newborn patients.

##### Randomization Unit

The FP will be considered the randomization unit for several compelling reasons.

1. The Italian National Health System mandates that all Italian newborns be assigned to an FP from birth until the age of 14 years. As a result, nearly all children were under the care of an FP, with a coverage rate exceeding 97%.
2. In Italy, FP is responsible for maintaining detailed and longitudinal health records of children under their care.
3. Utilizing the existing FP infrastructure for randomization is an economically efficient and practical approach. These healthcare providers are already embedded in the community and routinely interact with young patients, making them an accessible and efficient means of data collection and intervention implementation.
4. The Italian system for assigning newborns to FP has been in place since the early 1980s.

Before conducting any study procedures, FP will assess eligibility and seek informed consent from the parents or legal guardians of eligible children. The informed consent form can be found in Supplementary Materials.

##### Randomization Procedure

FP will be randomly assigned in a parallel 1:1 fashion to one of the two intervention groups. A block randomization procedure will be considered for the allocation of the FP to the intervention. To ensure unbiased allocation, this sequence will be concealed from the investigators. Investigative sites will access the randomization information of the enrolled participants through a dedicated online system via the RedCap (V 14.5.20) interface [29]. In line with ethical considerations, the enrolled participants and investigators will not be blinded to the study treatments, as the nature of the dietary intervention does not permit blinding. Additionally, participants will be asked for consent to share relevant data with researchers from participating universities or regulatory authorities where relevant.

The allocation sequence will be computer-generated by a statistician involved in the enrollment or assignment of participants. Participants will be enrolled by the research team, which may include healthcare providers and study coordinators at participating sites in Scampia. Following enrollment, a separate member of the research team who is not involved in the initial enrollment process will be responsible for assigning participants to interventions based on the pre-generated allocation sequence.

### 2.2. Intervention Details

The study arms are considered as follows:

- Intervention: Infants in the intervention group will follow a Mediterranean Diet-based weaning plan [14], starting with fresh purees (seasonal vegetables, blue fish) and flavored with herbs like thyme (*Thymus* spp.), parsley (*Petroselinum crispum*), and rosemary (*Rosmarinus officinalis*). Salt is excluded, and 2 g of parmesan cheese is added for tanginess.
- Control: Infants in the control group will follow a “traditional” weaning approach with industrial baby foods and delayed introduction of fresh ingredients.

The details of the intervention are reported in Table 3.

## 3. Outcomes

The primary study outcome is the incidence of obesity, celiac disease, and other conditions (measured annually using clinical assessments and BMI tracking), as reported in Table 4.

The secondary outcomes are as follows:

1. Microbiome composition: Fecal samples analyzed at 3- and 5-years using DNA sequencing. Evaluation of the fecal microbiome in children aged 3 and 5 years. Fecal samples will be obtained from study participants. These samples will then be analyzed in a laboratory setting, often using techniques such as DNA sequencing, quantitative PCR, or other microbiological assays. These methods enable the identification and quantification of specific bacterial species or groups as well as the detection of markers of inflammation.
2. Mediterranean Diet adherence: Measured annually using the KIDMED index. To quantify adherence to the Mediterranean Diet in children, the Mediterranean Diet Quality Index for Children and Adolescents (KIDMED) [30] will be considered. The KIDMED index assesses adherence to the Mediterranean Diet based on principles that underscore the intake of foods considered beneficial and the reduction of items that are not typical of the Mediterranean Diet.

Other details concerning the study timeline are reported in Supplementary Materials.

### 3.1. Endpoints

The primary endpoint is the Incidence Rate Ratio (IRR) in the treatment versus control groups (Table 1) from 1 to 6 years. It assesses disease incidence both in the general pediatric population and within at-risk subgroups (Table 1).

The secondary endpoints are as follows.

1. The difference in the rates of uninflamed microbiome in treatment versus control at six years of follow-up.
2. The 5-year rate of “High-Moderate” adherence in the Mediterranean Diet arm.

### 3.2. Statistical and Design Aspects

The PEMED trial is a cluster-randomized, parallel-group study using a Bayesian sequential design with a 1:1 allocation ratio. The randomization units for the experimental intervention will be FP. According to official statistics in Campania, there are 730 FP in the Campania Region (http://dati.istat.it/Index.aspx?QueryId=31546, accessed on 7 May 2024). Moreover, the city has a population of 911,222, of which approximately 10% (92,600 inhabitants) are residents of the Municipality Piscinola-Scampia. Recent statistics have indicated an average of approximately 800 patients per FP (www.istat.it, accessed on 7 May 2024).

Furthermore, the expected number of newborns in Municipality No. 8 for the year was 1179, with a population of 18,562 individuals aged 0–14 years in the considered area. The FP rate in the Piscinola-Scampia area was approximately 23 (18,562/800). Therefore, each pediatrician could have approximately 51 newborn patients per year (1179 newborns/23 FP).

Each FP will be randomized to an intervention. Following randomization, FP will direct the patients to the randomized intervention. The first interim assessment will be conducted at half of enrollment, that is, halfway through enrollment. If the intervention proves effective according to the statistical interim efficacy boundaries, the trial will be preliminarily stopped for efficacy; otherwise, the study will continue for up to 6 years (Supplementary Materials).

The study is designed conservatively, considering endpoints with lower frequencies, such as “Mental Retardation and Developmental Delay”, “Diabetes”, “Inflammatory Bowel Diseases”, “Cystic Fibrosis”, “Endocrinopathies”, “Nephro-Uropathies”. The expected number of events per year is 71 of 1179 newborns. Since there are approximately 70 FP, each specialist in the area would observe an average of $\mu =\frac{71}{23}=3.09$ cases per year.

The design proprieties are reported in a simulation experiment detailed in the Supplementary Materials.

#### 3.2.1. Sample Size

The sample size was derived via a simulation experiment, generating data in 1000 iterations. At each interim assessment, the posterior probability that the experimental intervention induced a reduction in the incidence of new cases in terms of the Incidence Rate Ratio (IRR) of at least 0.69 [31] was calculated as a proxy of empirical power. This value corresponds to a small effect size (Cohen’s d = 0.2) [32], as reported in the conversion formulas [33] (see the Supplementary Materials).

A total sample size of 23 FP with 51 patients followed per cluster would allow achieving a proportion of trials declared effective in at least 59% of the cases in scenarios with lower assumed overdispersion. This proportion increased to at least 81% during the second interim analysis. Across all planned simulations, the probability of erroneously declaring at least one intervention is effective when it is not (IRR = 1) and remains below 5%.

Simulations were conducted using R 3.4.1 [34].

#### 3.2.2. Statistical Analysis

Baseline data will be summarized as median (1st and 3rd quartiles) for continuous variables and percentages (absolute numbers) for qualitative variables.

Analysis of the primary endpoint of the study will be conducted using a Bayesian Group Sequential design with an expert elicitation procedure. The first interim analysis will be conducted when 50% of the study’s timing is reached.

The primary outcome will be assessed using a Bayesian sequential approach, with posterior probabilities used to evaluate intervention efficacy. Maternal and family characteristics, as well as physical activity data, will be incorporated as covariates to control for potential confounding effects on child health outcomes.

The trial’s findings, including primary and secondary outcomes, will be presented in future publications. Data will be analyzed using an Intention-to-Treat approach, with missing data imputed using a Last Observation Carried Forward (LOCF) protocol.

Other details about the statistical analysis and the design are reported in Supplementary Materials.

#### 3.2.3. Clinical Follow-Up and Outcome Assessment

Evaluation of clinical endpoints will be performed at specific time points. All newborns in the municipality will be automatically assigned to FP operating within the district. These Pediatricians initiate health surveillance for children starting from the first month of life, conducting health assessments that are documented in the dedicated logbook and recorded in the REDCap systems used by these healthcare professionals. During the initial registration of a child, the district informs the pediatrician of the child’s randomization status as either a case or control. Other Trial conductions details are reported in Supplementary Materials.

#### 3.2.4. Interim Analyses and Adaptive Adjustments

Interim analyses will be conducted at 50% enrollment using the Bayesian sequential framework to evaluate adherence to the Mediterranean Diet (Mediterranean Diet) and the intervention’s impact on obesity rates and microbiome composition. If low adherence is identified in specific participant subsets, targeted interventions such as additional counseling, tailored educational materials, or modifications to dietary guidance will be implemented to improve compliance.

## 4. Discussion

The present study holds promise both from a methodological perspective and in terms of its real-world impact, particularly within the challenging context of Scampia, a densely populated area in Naples known for its complex issues of social inclusion and poverty.

From a methodological standpoint, this study protocol considers a Bayesian Cluster randomized clinical trial sequential design, introducing an element of rigor to the research [35]. Moreover, the involvement of FP as the randomization unit is a distinctive feature of the study, capitalizing on Italy’s established system of assigning all newborns to FP until the age of 14 years [26]. This novel approach considers an existing data bank of longitudinal health information that is rarely utilized for epidemiological and clinical research purposes. By engaging the FP as the primary intervention unit, the study would maximize its potential to reach a large and diverse population of children while minimizing the need for new infrastructure or personnel [36].

This protocol establishes a framework for evaluating the impact of Mediterranean Diet-based nutrition on childhood health outcomes. The results, to be shared in future publications, are expected to guide public health strategies in underserved communities. Known for its cardiovascular and overall health benefits, the Mediterranean Diet, when introduced early, aims to foster lifelong healthy eating habits [37]. This pragmatic approach aligns with existing healthcare practices within Scampia, making it feasible and sustainable within the local healthcare system.

Moreover, a study is planned for evaluating a range of critical outcomes of not only health-related factors, such as microbiome status and disease incidence [38], but also sociodemographic elements such as nutritional choices and adherence to the Mediterranean Diet [39]. These assessments provide a holistic view of the impact of the intervention and its implications for child health and well-being.

This study design is aimed at being particularly tailored in the context of Scampia and other settings where social and economic disparities are prominent [40]. Working within the existing healthcare infrastructure has the potential to improve the health and quality of life of children in the community without imposing additional burdens on the system. Moreover, it would serve as a model for leveraging established healthcare networks to implement large-scale, cost-effective interventions in challenging socioeconomic environments [41].

The study’s methodological rigor and its adaptation to the peculiar real-world challenges of Scampia underscores its potential to generate useful results in child health, nutrition, and well-being. These findings are expected to contribute to the scientific literature and offer practical, scalable solutions for improving the health of vulnerable populations in similar contexts worldwide.

## 5. Conclusions

In conclusion, this manuscript outlines the protocol for the PEMED trial, which aims to improve child health and nutrition in real-world settings. The results of this study, to be presented in forthcoming publications, will provide critical results on the benefits of early adherence to the Mediterranean Diet. By considering the existing infrastructure of the FP system and promoting the Mediterranean Diet style of nutrition, this trial offers a cost-effective and pragmatic approach for instilling healthy dietary habits from an early age. A global assessment of health outcomes, including microbiome status and disease incidence, adds depth to our understanding of the impact of the intervention.

## Figures and Tables

**Table 1:** Child population with health needs in municipality 8, Scampia-Piscinola.

	Prevalence		Incidence	
	Population (0–18) = 28,915		Newborn (per Year) = 1179	Newborn (6 Years) = 7074
	Prevalence (×1000)	Expected Prevalent Cases over 28,915	Incident Cases/Year	Incident Cases/6 Years
Obesity	75	2169	88	528
Mental And Developmental Retardation	20	578	24	144
Congenital Malformations	6	173	7	42
Metabolic Diseases	0.8	23	1	6
Diabetes	1.2	35	1	6
Celiacy	20	578	24	144
Inflammatory Bowel Diseases	3.5	101	4	24
Cystic Fibrosis	0.8	23	1	6
Functional Bowel Disorders	35	1012	41	246
Growth And Nutrition Disorders	30	867	35	210
Endocrinopathies	11	318	13	78
Severe Asthma	20	578	24	144
Food And Skin Allergies	25	723	29	174
Nephro-Uropathies	12	347	14	84
Anaemia	13	376	15	90
Tumors	5	145	6	36
Total	278	8046	327	1962

**Table 2:** Maternal, family, and child factors collected during the study.

Variable	Category	Data Collected	Collection Time
Maternal Age	Maternal Demographics	Age of the mother at the child’s birth	At baseline (enrollment)
Maternal Education Level	Maternal Socioeconomic	The highest level of formal education attained	At baseline (enrollment)
Family Income	Family Socioeconomic	Reported household income level	At baseline (enrollment)
Maternal Smoking	Maternal Health Behaviors	Smoking status during pregnancy and breastfeeding	At baseline (enrollment)
Maternal Medication Use	Maternal Health	Use of prescribed or over-the-counter medications during pregnancy	At baseline (enrollment)
Family Socioeconomic Status	Family Environment	Composite measure of socioeconomic factors, including occupation	At baseline (enrollment)
Physical Activity (Children)	Child Behavior	Frequency and type of physical activities	During follow-up (yearly)
Dietary Habits (Family)	Family Behavior	Type of foods regularly consumed by the family	At baseline (enrollment)
Breastfeeding Duration	Child Nutrition	Length of exclusive and mixed breastfeeding	At baseline and follow-up (yearly)
Birth Weight	Child Health	Weight at birth	At baseline (enrollment)
Birth Length	Child Health	Length at birth	At baseline (enrollment)

**Table 3:** Overview of the intervention and control groups in the PEMED trial, highlighting key dietary components, exclusions, timeline, and implementation methods.

Aspect	Intervention Group (Mediterranean Diet)	Control Group (Standard Practice)
Dietary Approach	Mediterranean Diet (Mediterranean Diet) with fresh, seasonal foods (purees and mashed foods)	Standard weaning with industrial baby foods
Key Components	Seasonal vegetables (e.g., broccoli (*Brassica oleracea*), cauliflower (*Brassica oleracea* var. *botrytis*)), blue fish (e.g., anchovies, mackerel, and cod) flavored with herbs and spices	Delayed introduction of fresh foods and fish (after one year), predominantly processed baby food
Flavor Enhancers	Spices and herbs (e.g., thyme (*Thymus* spp.), rosemary (*Rosmarinus officinalis*), and parsley (*Petroselinum crispum*)); 2 g of parmesan cheese added for tanginess	None
Exclusions	No added salt, no sweets	None
Timeline	Starting at 5–6 months of age; adherence to Mediterranean Diet principles up to 3 years	Standard dietary practices as per regional guidelines
Delivery	Guidance and monitoring by Family Pediatricians (FPs)	Standard care provided by Family Pediatricians (FPs)

**Table 4:** Summary of primary and secondary outcomes in the PEMED trial, including the corresponding measurement methods and timing of assessments.

Outcome	Measurement Method	Timing
Primary Outcome		
Incidence of obesity, celiac disease, and other chronic conditions	Annual clinical assessments and BMI tracking	Years 1–6
Secondary Outcomes		
Microbiome composition	Fecal sample analysis (DNA sequencing, qPCR)	Years 3, 5
Mediterranean Diet adherence	KIDMED questionnaire	Annually
Growth parameters	Weight, height, BMI measurements	Annually
Markers of inflammation	Analysis of stool samples	Years 3, 5

## Data Availability

No new data are considered for the manuscript.

## Supplementary Materials

The following supporting information can be downloaded at https://www.mdpi.com/article/10.3390/jcm14010240/s1, Table S1. Scenarios characterizing the simulation experiment.
