# Supplementary material for: Bayesian Sequential Pragmatic Cluster Randomized Clinical Trial Design for PrEventive Effect of MEditerranean Diet in Children: PEMED Trial Research Protocol
Source: J Clin Med. 2025 Jan 3;14(1):240. doi: 10.3390/jcm14010240 (PMC11721821; doi:10.3390/jcm14010240)
Supplement: Supplementary file 1 [file jcm-14-00240-s001.zip › Appendix A Informed Consent Template (1) (1).pdf]

Informed Consent Form for Participation in the PEMED Clinical Trial

PEMED Trial - Impact of Mediterranean Diet on Health Outcomes in Children

Principal Investigator: Prof. Luigi Greco

You are being invited to participate in a clinical research study. Before you decide, it is important that you understand why the research is being done and what it will involve. Please take the time to read the following information carefully.

1. Study Overview: This study aims to evaluate the effects of the Mediterranean Diet (MD) on various health outcomes in children. Your participation will involve following specific dietary guidelines and attending regular health assessments.

2. What Will Happen in the Study: If you agree to participate, you will be randomly assigned to either the intervention group, receiving the MD, or the control group, following standard dietary practices. The study will monitor your health, growth, and dietary habits.

3. Voluntary Participation: Your participation in this study is completely voluntary. You are free to withdraw at any time without affecting your medical care.

4. Potential Risks and Benefits: While there are no known risks associated with following the MD, any unexpected reactions should be reported immediately. The potential benefits include improved health outcomes and nutritional habits.

5. Confidentiality: Your personal information will be kept confidential and will only be used for the purposes of this study.

6. Ethics Approval: This study has been reviewed and approved by the Ethics Committee, and follows the ethical guidelines of the World Medical Association Declaration of Helsinki.

7. Consent: I have read and understood the information above. I have had the opportunity to ask questions and have them answered satisfactorily. I understand that my participation is voluntary and that I am free to withdraw at any time, without giving a reason and without my medical care or legal rights being affected.

Participant's Name: ____________________________

Signature: ____________________________ Date: _______________

If you are signing on behalf of the participant:

Your Name: ____________________________

Relationship to Participant: ____________________________

Signature: ____________________________ Date: _______________
