# Supplementary material for: Bayesian Sequential Pragmatic Cluster Randomized Clinical Trial Design for PrEventive Effect of MEditerranean Diet in Children: PEMED Trial Research Protocol
Source: J Clin Med. 2025 Jan 3;14(1):240. doi: 10.3390/jcm14010240 (PMC11721821; doi:10.3390/jcm14010240)
Supplement: Supplementary file 1 [file jcm-14-00240-s001.zip › Appendix B statistical details_rev2DA (1).pdf]

Appendix B

Statistical Details

Simulation study and Design operative characteristics

The operational properties of the design were assessed through a simulation experiment with 1000 runs.

The data were sampled for each cluster (j) and each patient (i) from a quasi-Poisson distribution to account for potential overdispersion in the data:

$$Y_{ij} \sim QuasiPoi(\mu_0\,, \theta)$$
$$E(Y_{ij}) = \mu_0$$
$$\text{var}\,(Y_{ij}) = \theta E(Y) = \theta \mu_0$$

The intervention effect is defined for clusters randomized to the treatment as $\mu_1 = \mu_0 * IRR$.

The value $\sigma_w = \sqrt{\theta \mu_0}$ defines the within-cluster variability. Additionally, a term of between-cluster variability is added; thus, ${Y_{ij}}^* = Y_{ij} + \varepsilon_j$, where:

$$\varepsilon_j \sim N(0, \sigma_B)$$

Experiments were conducted under different scenarios. For each proposed scenario (Table 2), the data are simulated 1000 times, and for each simulation, the posterior probability is calculated for $P\left(\log\left(\widehat{IRR}\right) < 0\right) > \boldsymbol{\varphi}$, where $\boldsymbol{\varphi}$ represents the efficacy boundaries at the first Interim and the start of the study.

The posteriors were derived by analyzing the simulated data using a Markov Chain Generalized Linear Mixed Model (MCMC-GLMM) with 5000 iterations and four chains. By default, a normal distribution with a mean of zero and a very wide variance (1000) is assumed for fixed effects. For the variances of random effects, inverse Wishart priors were implemented, assuming no prior knowledge about the parameters $\nu = 0$. The model assumed a Poisson distribution for the outcome variable. The proposed parameterization accommodates a Poisson model while managing overdispersion without assuming a function to encapsulate this variability component, in contrast to the use of Quasi-Poisson estimation equations[1].

The efficacy boundaries are derived from the cumulative distribution function of a normal distribution defined on the O'Brien and Fleming frequentist boundaries to translate the efficacy limits into a Bayesian approach, as suggested in the literature[2]. The boundaries are $\varphi_{(z=2.8)}^{interim} = 0.997$ and $\varphi_{(z=1.98)}^{overall} = 0.976$.

Within each scenario, the proportions of trials correctly declared the intervention efficacy and the first and second interim analyses were evaluated as proxies for the empirical power of the study.

The evaluated scenarios are as reported in the Table S1

The results of the simulation experiment are presented, showing the proportion of trials correctly declared effective at the first and second interim analyses (Figure S1).

Table S1 *Scenarios characterizing the simulation experiment*

Simulation Parameter	Values
**Number of Clusters (FP)**	23
**Number of patients per FP per year**	50
**Timing First Interim**	3 years
**Timing Second Interim**	6 years
$\boldsymbol{\sigma_B}$ **(variance between)**	0.2-0.5
**IRR**	0.71
**Number of expected events per year per cluster**	3.09/year
**Dispersion Parameter** $\boldsymbol{\theta}$	1-2

Statistical Analysis

The chosen model for evaluating the IRR on the primary endpoint will be defined by a Bayesian MCMC-GLMM model. Priors will be defined through a SHELF (Sheffield Elicitation Framework) elicitation paradigm[3]. This method involves interaction between a facilitator and a group of 5 experts who will collaborate to define a prior probability distribution on the probability of remission. The procedure will be repeated until the pooling of expert opinions satisfies all parties involved in the elicitation process. The elicitation procedure will be conducted using R Shelf software[4]. Non-informative priors will still be used for sensitivity analyses.

Upon reaching the interim analysis, the probability that the posterior probability of $\log(\widehat{IRR})$ is less than one will be calculated and compared to the efficacy boundaries defined in the interim, as stated in the study design section. If $P(\log(\widehat{IRR}) < 0) > \varphi^{interim}_{(z=2.8)}$, then the trial will terminate for efficacy; otherwise, the trial will continue until its completion.

If the trial continues, the posterior probabilities for $\log(\widehat{IRR})$, with 95% credibility intervals, will be calculated, and the intervention will be declared effective if $P(\log(\widehat{IRR}) < 0) > \varphi^{overall}_{(z=1.98)}$.

Secondary analyses will be conducted using generalized Bayesian models, with interventions as covariates, and 95% credibility intervals on the posteriors will be reported. The posterior probabilities with credible intervals will be also considered for the secondary endpoints.

Model convergence will be assessed using trace plots and Gelman statistics. Data will be analyzed using an Intention-to-Treat approach, and missing data will be imputed according to a Last Observation Carried Forward (LOCF) protocol.

Sheffield Elicitation Framework of Expert Opinion

A group of five subject-matter experts will be carefully chosen based on their expertise relevant to the trial's focus, such as pediatric nutrition, epidemiology, and biostatistics.

A trained facilitator will guide the elicitation process. This facilitator will be responsible for steering discussions, clarifying concepts, and ensuring that each expert's opinion is adequately considered and integrated.

The SHELF[5] methodology provides a structured approach for expert elicitation. It involves a series of steps designed to facilitate the gathering and synthesizing of expert opinions into a coherent prior probability distribution.

- Experts will initially provide their assessments regarding the probability of remission based on their knowledge and experience.
- Following individual assessments, a group discussion will be facilitated to explore different perspectives, reconcile differences, and seek a consensus.
- Experts will have the opportunity to revise their initial assessments based on the group discussion.
- The process will be repeated iteratively until a consensus is reached among all experts.

The R Shelf[4] software will be used to assist in the elicitation process. This tool helps in organizing and visualizing expert input, facilitating the synthesis of individual assessments into a collective prior distribution. The entire process, including individual and group assessments, rationales behind opinions, and the final consensus, will be thoroughly documented.

To validate the robustness of the trial findings, non-informative priors will also be utilized in sensitivity analyses alongside the expert-defined priors.

Procedures for Handling Missing, Unused, or Spurious Data
All analyses will be conducted based on the available data, and missing data will be excluded from the analysis. Any unused or spurious data will be appropriately noted in the final report. To identify potential important influences on the study design due to missing values, analyses will also be compared using a multiple imputation approach.

Deviations from the Original Statistical Plan
Any significant deviations from the original statistical plan outlined above will be thoroughly documented in an amendment to the clinical investigation plan. Minor changes to the planned analyses will be detailed in the final report.

Public Access Policy
The complete protocol, participant-level dataset, and statistical code may be shared or made publicly available for specific purposes or upon request by participants, subject to approval by the steering committee. The datasets analyzed during the current study can be obtained from the corresponding author upon reasonable request.

References

1. Hadfield JD. MCMC Methods for Multi-Response Generalized Linear Mixed Models: The MCMCglmm R Package. *Journal of Statistical Software* 2010; 33: 1–22.

2. Yin G, Shen Y. Adaptive design and estimation in randomized clinical trials with correlated observations. *Biometrics* 2005; 61: 362–369.

3. O'Hagan A, Buck CE, Daneshkhah A, et al. *Uncertain judgements: eliciting experts' probabilities*. John Wiley & Sons, 2006.

4. Oakley J. *SHELF: Tools to Support the Sheffield Elicitation Framework*, https://CRAN.R-project.org/package=SHELF (2019).

5. Gosling JP. SHELF: The Sheffield Elicitation Framework. In: Dias LC, Morton A, Quigley J (eds) *Elicitation*. Cham: Springer International Publishing, pp. 61–93.
