# Supplementary material for: Bayesian Sequential Pragmatic Cluster Randomized Clinical Trial Design for PrEventive Effect of MEditerranean Diet in Children: PEMED Trial Research Protocol
Source: J Clin Med. 2025 Jan 3;14(1):240. doi: 10.3390/jcm14010240 (PMC11721821; doi:10.3390/jcm14010240)
Supplement: Supplementary file 1 [file jcm-14-00240-s001.zip › Appendix C Other trial details (1) (1).pdf]

Appendix C

Other trial organizational details

Study Timeline and Implementation Steps

The study involved a total duration of six years, with the project intervention, including dietary changes, spanning the initial three years. The subsequent 3 years will be dedicated to monitoring outcomes. It should be noted that participant recruitment will occur over 3 years, and each individual's participation will extend for 3 years.

1. **Time 0-2 Months**: An explanatory meeting with the FP (Family Pediatrician) will be held to enhance their daily work and propose quality control measures for health assessment documentation. Any potential simplifications or additional improvements to the health assessment modules managed by the pediatricians' computerized systems will be analyzed. Communication materials for families are prepared. The office will be strengthened for the monthly collection of health assessment forms, considering the possibility of electronic data transmission from pediatricians to the district, and potential reporting to pediatric specialists.
2. **Time 2 Months and Beyond** Dietary support will be activated for both randomized families and controls, and preparations will be made for the collection of fecal and salivary samples
3. **Time 2-6 Months**: Identification of subjects at familial risk will be conducted through appropriate modifications to the initial health assessment, particularly in the first month of life.
4. **Time 3:2-6 Years**: Activation of the specialist consultancy service for children referred by Pediatricians to the Network of Project Specialists. This includes the consideration of transitioning these cases to the Pediatric University Health Center of Scampia along with the corresponding diagnostic and therapeutic pathways.
5. **Time 4**: Continuous monitoring of the project progress, interim analysis of the collected data, and dissemination of results to pediatricians.
6. **Time 5**: Comprehensive analysis and dissemination of study results, as well as closure of the randomization phase at 3 years.

Electronic data collection forms will be accessible in e-CRF REDCap[1]. FP at each eligible patient will receive preliminary training from the coordinating center to ensure the quality of both clinical data and the recording of clinical events.

Organization and Ethics

Study Organization

The study, known as PEMED, was sponsored by the Department of Translational Medicine of Federico II University, located in Naples, Italy. The steering committee of PEMED assumes full responsibility for the study's design, implementation, data analysis, and preparation of all manuscripts, including the present

one. Access to the final study dataset, which includes actual patient data and statistical programming, was granted exclusively to the steering committee.

The committee is composed of Luigi Greco (UNINA), Danila Azzolina (UNIFE), Renata Auricchio (UNINA)

A clinical endpoint committee was established to adjudicate primary endpoint events and assess secondary safety events. A data safety monitoring committee will conduct safety monitoring to ensure the safety and integrity of this study. The committee participants were the same as those of the Steering Committee.

In the PEMED trial, the interim was designed to assess early data on efficacy, safety, and other critical metrics. Guidelines have been established based on specific criteria, such as unexpected safety concerns or overwhelming evidence of efficacy. Access to interim results will be restricted to an independent data-monitoring committee tasked with reviewing the data and ensuring objectivity. This committee is responsible for making recommendations based on interim analyses.

Monitoring and quality

A designated coordinating investigator or their designees will conduct monitoring activities throughout the study following a predefined monitoring plan. Monitoring visits will be scheduled at appropriate intervals to review the FP data for accuracy and completeness, ensuring protocol compliance. The study monitor may inspect all relevant documents and records maintained by the FP and sites, including medical records (office, clinic, or hospital) for study subjects. FP are obligated to grant access to such records. Source documentation must be available to validate informed consent procedures, adherence to protocol procedures, accurate reporting, and follow-up data accuracy on case report forms.

Initial monitoring will be conducted approximately two weeks after the first patient's inclusion, followed by subsequent visits every 12 months at the FP. The monitoring schedule may be adjusted based on factors such as subject enrollment rates, quality concerns, site compliance, or other site-specific issues. Any significant deviation from the planned monitoring timelines will be documented in the monitoring visit report, and the monitoring plan will be amended if deemed necessary.

A comprehensive schedule of well-baby clinical check-ups was established to monitor and assess adherence to the Mediterranean Diet. These checkups will occur at key developmental stages, including 6, 12, 16, 18, and 24 months of age, and will be conducted by the FP. Additionally, a professional dietitian will closely monitor adherence every three months up to the age of 24 months.

The control group will follow the standard clinical routine. The control arm considers the following:

- Standard guidance on breastfeeding, weaning, and nutrition during the first 1000 days is in line with the Regional Guidelines.
- Quarterly dietary surveillance and health balance sheets

For each child, whether in the case or control group, after obtaining Informed Consent, we will offer the opportunity for saliva collection via a small swab at 6 months and 3 years. Additionally, we requested stool samples at 3 and 5 years. These samples will be stored in a Biobank and subjected to genetic risk factors and microbiome analysis at Federico II Laboratories for microbiome analysis at the Department of Agriculture.

In the PEMED trial, the allocated interventions will be discontinued or modified if participants experience adverse dietary reactions or upon the request of participants or their guardians. Additionally, significant changes in a participant's health status prompted adjustments to the intervention. Decisions regarding these modifications will be made in collaboration with healthcare professionals to ensure the safety and well-being of participants.

Adherence monitoring will be conducted through periodic dietary recall questionnaires and, if feasible, through analysis of biomarkers in blood samples to assess nutritional compliance. Regular follow-up appointments with healthcare professionals will also be scheduled to reinforce adherence and address any concerns or obstacles faced by the participants.

During the PEMED trial, participants will be allowed to receive standard medical care and any necessary treatment unrelated to the trial interventions. However, the introduction of additional dietary supplements or major changes in dietary habits outside the trial protocols will be prohibited to maintain the integrity of the study. Regular health checkups and vaccinations, as per the pediatric guidelines, will be permitted and encouraged. Any concomitant care or intervention that could potentially interfere with the study's outcomes will be closely monitored and assessed for compatibility with the trial's objectives.

Data

The PEMED trial will be conducted in a community setting, specifically Scampia, a municipality in Naples, Italy. This setting will involve collaboration with local family pediatricians, integrating the study into existing community healthcare infrastructure. As the trial is localized, all data collection will occur within Italy, focusing on the unique socioeconomic and cultural context of Scampia. Data entry will be conducted using a web-based electronic data capture system, with access granted via individual usernames and passwords issued to each site's principal investigator or designees. Each study subject will be identified by a numeric code derived from the study site code and progressive enrollment code. To ensure data quality, the e-CRF system incorporates range checks for the data values. Routine data quality assessments will be conducted at specified intervals as outlined in the monitoring plan. These assessments will encompass a centralized data quality monitoring system as well as manual data quality checks to address data queries with investigators and collaborators at each study site. The subjects' privacy rights will be safeguarded in accordance with the EU General Data Protection Regulation (GDPR). The detailed data management procedures are outlined in a separate document (e-CRF manual). Baseline data, including demographic information, medical history, and initial dietary habits, will be collected at the outset. Outcome data, such as growth parameters and fecal microbiome analysis, will be gathered periodically. To ensure data quality, key measurements will be performed twice, and the assessors will undergo specialized training. The trial will utilize questionnaires for dietary adherence and laboratory tests for nutritional and microbiome analysis. Data collection forms, if not included in the main protocol, will be accessible through an online repository or the trial's coordination center.

The data will be securely entered and coded into a password-protected database with anonymity maintained through coding. Strict access controls ensure data security and regular backups prevent data

loss. All electronic data will be stored on encrypted servers, and any physical document is stored in a secure location.

The FP will retain all records related to this study for a period of three years following the conclusion of the study, as directed by the coordinating investigator (or their designees), or in compliance with local regulations if stipulating a longer retention period.

Patient retention and protocol deviation data

To promote participant retention and ensure complete follow-up in the PEMED trial, a comprehensive plan will be implemented. This plan will include regular communication with participants and their families, offering flexible scheduling for assessments to accommodate their needs, and providing continuous education and support regarding the importance of the study. Additionally, motivational incentives may be used to encourage ongoing participation.

The trial will still aim to collect key outcome data from participants who discontinue or deviate from the intervention protocols. This includes measurements, such as growth parameters, incidence of relevant health conditions, and dietary habits at the point of discontinuation.

Adverse reaction data

Adverse diet reactions will be documented in a standardized manner, and their severity and potential relationship with the trial interventions will be evaluated. All events will be promptly reported to the trial oversight committee. Regular reviews of the reported adverse reactions will be conducted to identify any patterns or safety concerns that may arise during the trial.

Patient Compliance and Follow-Up

FP are encouraged to employ strategies to enhance patient compliance with the study protocol and treatment. During follow-up visits, the FP actively monitored adherence to the Mediterranean Diet through a dedicated questionnaire integrated into the e-CRF.

Recruitment

The PEMED trial will employ targeted outreach, collaboration with local healthcare providers, and community engagement initiatives to achieve adequate participant enrollment and reach the target sample size.

Ethical Considerations

All subjects will be required to provide written informed consent, following the guidelines of the Ethics Committee (ECs) and utilizing EC-approved informed consent forms. Any additional individuals mandated by the site's EC to sign the informed consent form must adhere to this requirement. The principles outlined in the World Medical Association Declaration of Helsinki: Ethical Principles for Medical Research Involving Human Subjects should be strictly adhered to, ensuring that all subjects are fully informed.

Any significant changes to the protocol will be promptly communicated to all relevant parties including investigators, REC/IRBs, trial participants, trial registries, journals, and regulators. The research team will

be responsible for obtaining informed consent or assent from the participants or their authorized surrogates, ensuring a clear understanding of the trial.

If ancillary studies are planned, additional consent will be sought from the participants for the use of their data and biological specimens.

Personal information about the participants will be collected, shared, and maintained with strict confidentiality measures throughout the trial to protect privacy.

The financial and other competing interests of the principal and site investigators will be disclosed to maintain transparency. At the time of writing protocol no finantial disclosure are to declare

The steering committee will have access to the final trial dataset, including any restrictions owing to contractual agreements.

The investigators plan to disseminate the trial outcome results to participants, healthcare professionals, the public, and other relevant groups through publications or other data-sharing arrangements at the time of interim analysis and final study assessment.

The members of the steering committee, who are part of the authorship of the trial, will be joined by other experts, as deemed appropriate by the study's scientific board.

References

1. Harris PA, Taylor R, Minor BL, et al. The REDCap consortium: Building an international community of software platform partners. *Journal of Biomedical Informatics* 2019; 95: 103208.
